# Exercise Training after Myocardial Infarction Attenuates Dysfunctional Ventricular Remodeling and Promotes Cardiac Recovery

**DOI:** 10.31083/j.rcm2304148

**Published:** 2022-04-19

**Authors:** Shuqing Liu, Xinxiu Meng, Guoping Li, Priyanka Gokulnath, Jing Wang, Junjie Xiao

**Affiliations:** ^1^Institute of Geriatrics (Shanghai University), Affiliated Nantong Hospital of Shanghai University (The Sixth People’s Hospital of Nantong), School of Medicine, Shanghai University, 226011 Nantong, Jiangsu, China; ^2^Cardiac Regeneration and Ageing Lab, Institute of Cardiovascular Sciences, Shanghai Engineering Research Center of Organ Repair, School of Life Science, Shanghai University, 200444 Shanghai, China; ^3^Cardiovascular Division of the Massachusetts General Hospital and Harvard Medical School, Boston, MA 02114, USA

**Keywords:** exercise training, ventricular remodeling, microRNA, myocardial Infarction

## Abstract

Recent evidences have shown that exercise training not only plays a necessary role in maintaining cardiac homeostasis, but also promotes cardiac repair after myocardial infarction. Post-myocardial infarction, exercise training has been observed to effectively increase the maximum cardiac output, and protect myocardial cells against necrosis and apoptosis, thus leading to an improved quality of life of myocardial infarction patients. In fact, exercise training has received more attention as an adjunct therapeutic strategy for both treatment and prevention of myocardial infarction. This review summarizes the experimental evidence of the effects of exercise training in ventricular remodeling after myocardial infarction, and tries to provide theoretical basis along with suitable references for the exercise prescription aimed at prevention and therapy of myocardial infarction.

## 1. Introduction

Cardiovascular disease (CVD) has become one of the most common causes of human mortality throughout the world [1]. Aging populations, fast paced modern lifestyle, poor dietary habits and other socio-psychological factors, are leading towards a constantly and rapidly increasing risk for CVD in young and low-income population [2]. Myocardial infarction, which is one of the most common causes of mortality among CVDs, occurs when narrowing coronary arteries are blocked due to blood clot, cholesterol or fat deposits that prevent blood from flowing into the heart [3, 4]. During myocardial infarction, the blockage of blood flow to a part of the heart leads to an insufficiency of oxygen in the myocardium [5, 6]. Consequently, the left ventricular wall of the heart becomes thinner and dilates, causing decreased ejection fraction, and finally, the myocardial injury area is filled with scar tissue without any diastolic or systolic functions. Patients with severe myocardial infarction are likely to develop heart failure [7, 8]. The prognosis of acute myocardial infarction is closely associated with the size of the infarct area. Without early effective treatment, myocardial infarction will lead to continuous deterioration of the disease process and even death [9]. At present, the most commonly used method for effectively reducing myocardial ischemic injury is the reperfusion therapy [10, 11]. However, reperfusion therapy often causes reperfusion injury and triggers ventricular remodeling [11].

For a long time, exercise training was considered as a significant part in maintaining cardiovascular health. It is reported to be an effective intervention for both primary and secondary prevention of cardiovascular diseases in many clinical studies [12, 13, 14, 15]. Regular exercise training can increase coronary blood flow by improving vasodilatory functions, thereby reducing myocardial oxidative stress, preventing myocardial cell loss and limiting cardiac fibrosis, which in turn reduce the risk of coronary heart disease, myocarditis, myocardial infarction, and other cardiovascular diseases [16, 17].

Additionally, *in vivo *experiments revealed exercise training could delay cardaic aging and reduce aging-related cardiac fibrosis, apoptosis, and necrosis [18, 19]. Recent research by several groups have uncovered that exercise training could significantly reduce the occurrence of myocardial ischemia and reperfusion injury, offer protection from dilated cardiomyopathy and hypertrophic cardiomyopathy, by increasing the activity of endothelial nitric oxide synthase (eNOS) - nitric oxide (NO) and phosphoinositide 3-kinase (PI3K) signaling pathways [12, 20, 21, 22, 23]. Moreover, exercise training was verified to be able to attenuate ventricular remodeling after myocardial infarction [24, 25]. Thus, exercise training is increasingly receiving more attention in the context of both prevention and treatment of cardiovascular diseases.

## 2. Ventricular Remodeling Triggered by Myocardial Infarction

When the ventricular remodeling occurs, mechanical, neurohormonal, or genetic factors would alter the shape, size and function of the ventricles [26, 27]. The ventricular volume overload suddenly increases, triggering the process of remodeling in the infarcted area after acute myocardial infarction [28]. Myocardial hypoxia leads to an increased activation of neurohormones by inducing the migration of immune cells such as neutrophils, monocytes and macrophages to the infarct area, resulting in local inflammation [29, 30]. One of the key processes in post-infarcted remodeling is inducing cardiomyocyte hypertrophy [31]. Myocardial hypertrophy counteracts the increase in ventricular volume after myocardial infarction, weakening the progressive expansion of the myocardium, and stabilizes the myocardial contractile function [32, 33]. Therefore, cardiomyocyte hypertrophy is initially an adaptive and a protective response to the pathological change of myocardial infarction. However, at later stages various paracrine and autocrine factors, chronic neurohormonal activation, renin-angiotensin-aldosterone system activity (RAAS), and myocardial stretching, would continue to stimulate eccentric pathological hypertrophy, gradually leading to left ventricular failure [34, 35, 36].

Myocardial infarction also increases the degree of oxidative stress [37]. Low concentration of reactive oxygen species (ROS) is known to play an important role in signal transmission. However, higher ROS concentrations can directly impair cell membrane lipids, nuclear and mitochondrial deoxyribonucleic acid (DNA), as well as proteins thus causing severe and fatal cellular damage [38]. In fact, myocardium of congestive heart failure patients was found with excessive oxidative stress [39]. These observations indicated that a damaged antioxidant system and/or enhanced reactive oxygen species could elevate oxidative stress, resulting in dysfunction and poor remodeling of the infarcted myocardium. In addition, the growth of new capillaries and small arteries after myocardial infarction, or the occurrence of angina pectoris, were key processes of ventricular remodeling [40, 41, 42]. Damaged angiogenesis may lead to maladjusted left ventricular remodeling and promote transition from adaptive cardiac hypertrophy to left ventricular dilation and dysfunction [43, 44].

## 3. Improved Ventricular Remodeling Caused by Exercise Training Following Myocardial Infarction

The heart tends to be hypertrophic following stress stimulation, which is generally classified into either physiological hypertrophy or pathological hypertrophy. These two types of cardiac hypertrophy have significant differences in structure, function and molecular mechanism [31, 45]. Pathological hypertrophy is often accompanied by myocardial fibrosis, myocardial cell apoptosis and necrosis, and eventually develops into heart failure [31, 40]. However, physiological hypertrophy is an adaptive response induced by long-term standardized exercise training, which does not result in adverse remodeling including myocardial fibrosis. Unlike pathological hypertrophy, physiological hypertrophy is found with a protective effect on the heart [46].

Aerobic exercise training for eight weeks after undergoing surgery for myocardial infarction was revealed to increase the cardiac function in rats with chronic heart failure (CHF), accompanied by reduced cardiac remodeling, left ventricular end-diastolic pressure (LVEDP), left ventricular hypertrophy, and left ventricular collagen volume fraction. These changes could also help reduce the congestion of lungs [25, 47]. Similarly, exercise training programs reduced the degree of inflammation in myocardium, which indicated that physical exercise played a key role in controlling chronic systemic inflammation observed during heart failure [48, 49, 50]. In another exercise training model involving swimming in rats, researchers observed that compared to the infarct group without exercise training, exercise training reduced left ventricular expansion and thickened the non-infarct wall [24]. Exercise training was also observed to limit undesirable remodeling by weakening ventricular dilation and reducing wall tension in animal models having left ventricular dysfunction post-myocardial infarction [25, 51, 52]. Additionally, following exercise training abnormal expression of β-myosin was also found to be decreased [53].

Studies have demonstrated that free-wheeling exercise had little effect on the left ventricular geometry and function in mice from the sham-operated group. However, in severe myocardial infarction surgical group, free-wheeling exercise training was able to limit a further increase in post myocardial infarction mortality as well as simultaneously improve left ventricular remodeling, capillaries, and distal myocardial hypertrophy in this group. Moreover, the myocardial interstitial fibrosis and apoptosis were observed to be reduced after exercise training [54].

## 4. Protective Mechanisms Involved in Ventricular Remodeling due to Exercise Training post —Myocardial Infarction

Myocardial infarction has been accompanied by a variety of processes that lead to heart function damage, including reduced myocardial contractility, unbalanced energy metabolism, increased oxidative stress, escalated apoptosis, altered myocardial microstructure, and rapidly surging inflammatory response [55, 56, 57, 58, 59]. Exercise training protects ventricular remodeling and cardiac function through the following mechanisms: (1) regulation of the expression of certain microRNAs (2) adjustment of cardiac function either by improving the balance between metallopeptidase inhibitor 1 (TIMP-1) and matrix metalloproteinase-1 (MMP-1), thereby enhancing myocardial contractility; or by adjusting collagen accumulation to reduce cardiac rigidity and promote myocardial contractility (3) by regulating the energy metabolism of myocardial cells, such as increasing the level of catecholamines in local areas and in blood, increasing plasma free fatty acid (FFA) levels, increasing mitochondrial synthesis, and increasing adenosine triphosphate (ATP) production (4) through inhibition of oxidative stress of cardiomyocytes by activating PI3K-protein kinase B (PI3K-Akt) signaling pathway, increasing endothelial nitric oxide synthase (eNOS) activity and nitric oxide (NO) production in vascular endothelial cells (5) by enhancing vascular endothelial growth factor (VEGF) dependent angiogenesis pathways through increase in vascular shear stress, including increasing coronary vascular network and density, increasing myocardial blood flow perfusion signals, promoting angiogenesis, and thereby regulating ventricular remodeling (6) by increasing immunosuppressive factor interleukin 10 (IL-10), inhibiting expression of inflammatory factors such as tumor necrosis factor alpha (TNF-α) and interferons alpha (IFN-α), and regulating inflammatory response in the heart [60, 61].

### 4.1 Regulation of miRNA Expression in Cardiac Tissue

MicroRNAs (miRNAs, miRs), regulate protein translation via regulating the stability of messenger RNA (mRNA) to modulate numerous signaling pathways and cellular processes. MiRNAs have been reported to regulate cell-to-cell communication by altering the expression of signaling molecules involved in key biomolecular processes [62, 63]. In fact, the study of miRNAs in the context of cardiovascular pathophysiology could provide a new perspective, as they have been observed to play a key role in patients with CVD, such as myocardial infarction, hypertrophy, fibrosis, heart failure, arrhythmia, inflammation and atherosclerosis [64, 65]. MiRNAs have also been shown to regulate important processes that can lead to the pathophysiological consequences of acute myocardial infarction, by regulating cardiomyocyte apoptosis, and the formation of new blood vessels after ischemia [66, 67, 68, 69, 70]. Cardiac regeneration is also affected by miRNAs that control cardiomyocyte proliferation. In addition, miRNAs could also directly reprogram myocardial fibroblasts into cardiomyocytes to regenerate injured myocardium [71, 72].

MiRNAs can not only be used as important targets in the treatment of CVD, but also as important biomarkers indicative of systemic functionality [73, 74]. Exercise training was reported to induce changes in specific miRNAs expression levels in heart tissue. In addition, specific circulating miRNAs were observed to be expressed in response to exercise training, along with their corresponding downstream signals [75, 76]. Thus, the heart function could be regulated either by knocking down or over-expressing of these miRNAs [77, 78, 79].

The first miRNAs that were found and studied in exercise training animal models are three heart-specific miRNAs namely miR-1, miR-133a, miR-133b. In two independent experimental groups, swimming training and interval training of rats, the expression of these miRNAs was downregulated in heart tissues. Another kind of miRNA, highly expressed miR-21 was observed in cardiac fibroblasts during acute myocardial infarction as well as transverse aortic constriction (TAC) and enhanced the mitogen-activated protein kinase-extracellular signal-regulated kinase (MAPK-ERK) signaling pathway by inhibiting false homolog 1 (Spry1) [80]. In another study, myocardial infarction decreased the expression of miR-1 and increased the expression of miR-214. It has been reported that exercise training could prevent myocardial infarction induced reduction of miR-1 expression and increased miR-214 expression. These responses may be associated with the normalization of ${\mathrm{Ca}}^{2+}$ handling and left ventricular compliance in infarcted hearts due to exercise training, thereby promoting cardiac recovery [77, 81, 82].

Exosomes released during exercise training were proved to contain microRNAs—miR-455, miR-29b, miR-323-5p, and miR-466 that bind to the 3’ region of matrix metallopeptidase 9 (MMP9) and downregulates its expression, thereby reducing its harmful effects. Among these miRNAs, miR-29b and miR-455 have shown the highest regulation. On comparison with the non-exercise group, MMP9 activity of the exercise group was significantly reduced [83]. A study with aerobic training using animal model demonstrated that aerobic training could promote an increase in miR-126 expression by indirectly regulating VEGF pathway and directly regulating the mitogen-activated protein kinase (MAPK) and PI3K-Akt-eNOS pathways, which are associated with exercise-induced cardiac angiogenesis [84]. Single left ventricular myocyte dimensions were increased while cell-contraction and relaxation became faster during resistance training. These mechanical adaptations were corelated with the overexpressed expression of sarco/endoplasmic reticulum ${\mathrm{Ca}}^{2+}$-ATPase 2alpha (SERCA2α), which in turn, has effects in epigenetic modification of decreased miR-214 expression [85]. In addition, miR-17-3p protected against myocardial ischemic-reperfusion injury by metalloproteinase inhibitor 3(TIMP3) and phosphatase and tensin homolog-protein kinase B (PTEN-Akt) pathway which contributed to exercise-induced cardiac growth [25]. Another study found that aerobic training increased miR-29 expression and correspondingly reduced collagen expression levels in heart, resulting in improved left ventricular compliance and had beneficial cardiac effects. This protective effect was verified to be associated with high-performance aerobic training [78, 79] (Table 1, Ref. [25, 71, 78, 79, 80, 81, 82, 83, 84, 85]).

**Table 1.** **Summary of miRNAs regulated during exercise training with a protective role against heart diseases**.

Diseases	Exercise	Targets	miRNAs	Regulation Function	References
MI	Running	-	miR-1 ↑	${\mathrm{Ca}}^{2+}$ handling diastolic function ↑	[71, 81, 82, 85]
		SERCA2α	miR-214 ↓		
AMI/TAC/IRI	Swimming	Spry1	miR-21 ↑	cardiac fibroblasts; cardiomyocyte apoptosis ↓	[80]
-	Swimming/Running	MMP9	miR-455, miR-29b, miR-323-5p, miR-466 ↓	fibrosis ↓	[83]
-	Swimming/Running	MAPK&PI3K-Akt-eNOS pathways	miR-126 ↑	angiogenesis ↑	[84]
IRI	Swimming	TIMP3&PTEN-Akt pathway	miR-17-3p ↑	myocyte proliferation ↑	[25]
Ventricular compliance	Swimming/Running	Collagen gene	miR-29 ↑	cardiac fibroblasts ↓	[78, 79]

MI, Myocardial Infarction; AMI, Acute myocardial infarction; TAC, Transverse Aortic Constriction; IRI, Ischemia/Reperfusion Injury; MAPK, Mitogen-Activated Protein Kinase; MMP9, Matrix Metallopeptidase 9; PTEN, Phosphatase and tensin homolog; eNOS, Endothelial Nitric Oxide Synthase; TIMP3, Recombinant Tissue Inhibitors of Metalloproteinase 3; Akt, Protein Kinase B; PI3K, Phosphatidylinositol-3-Kinase.

### 4.2 Altering Myocardial Contractility

**Fig. 1.**
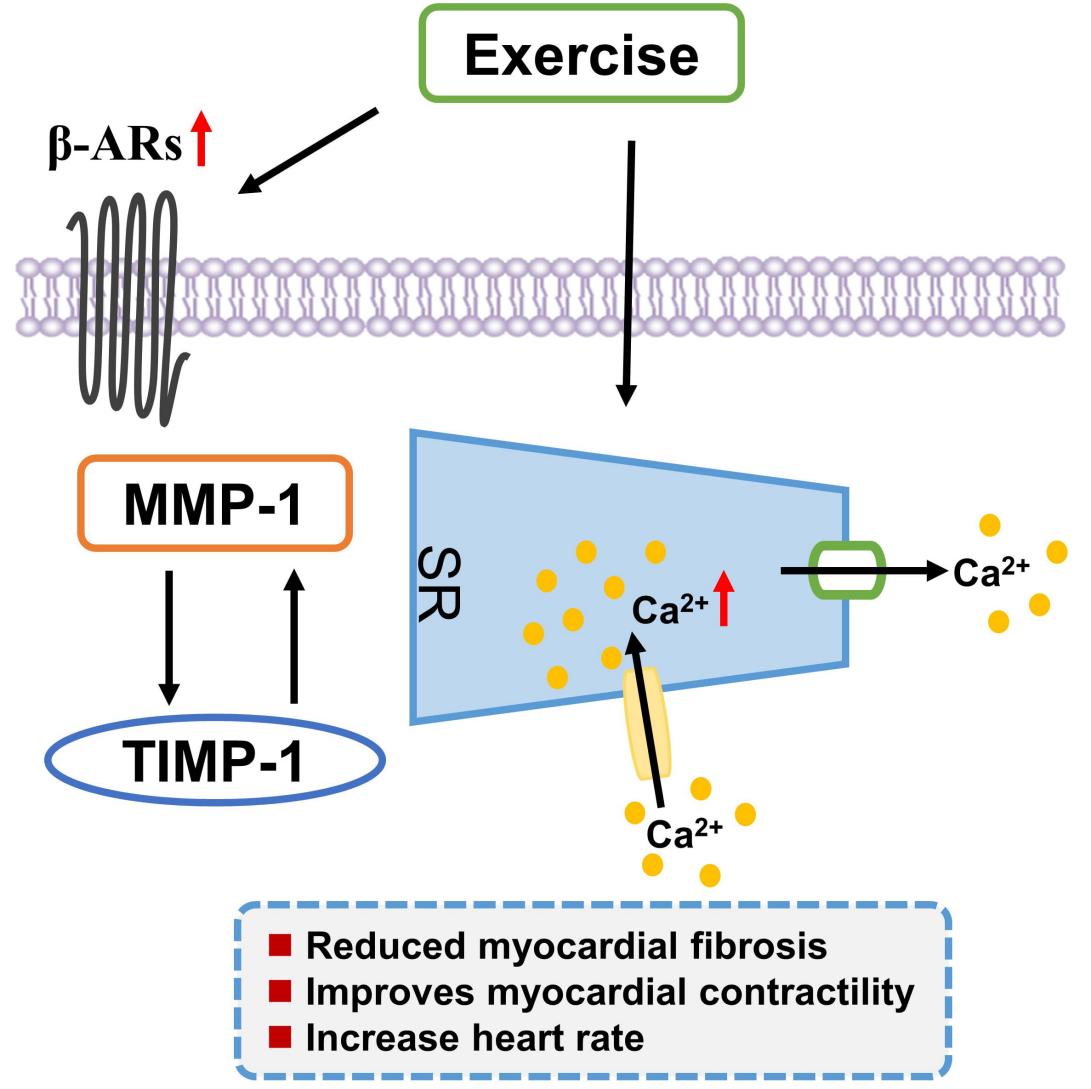
**Exercise training positively regulates calcium homeostasis, improves the balance between MMP-1 and TIMP-1, and enhances the expression of β-adrenergic receptors**. MMP-1, matrix metallopeptidase 1; TIMP-1, tissue inhibitor of metalloproteinases 1; SR, sarcoplasmic reticulum; β-ARs, β-adrenergic receptors.

After myocardial infarction, the infarct myocardium becomes composed of scar tissue without systolic and diastolic function [86, 87]. The contractile and diastolic ability of the heart muscle is greatly reduced. Exercise training can reduce myocardial fibrosis [81, 86]. Exercise training can also improve the balance between matrix metallopeptidase 1 (MMP-1) and tissue inhibitor of metalloproteinases 1 (TIMP-1), thereby reducing the stiffness of the heart by regulating collagen accumulation [18, 88, 89, 90, 91]. Studies have demonstrated that exercise training notably improves β-adrenergic receptors (β-Ars), reverses the major histocompatibility complex (MHC) α-β-cardiac isotype transition, and improves myocardial contractility [20, 92] (Fig. 1).

Exercise training can also improve the myocardial contractility by increasing cardiac ${\mathrm{Ca}}^{2+}$ intake by targeting SERCA2a or SERCA2a regulators/modifiers after myocardial infarction [93, 94]. The expression of SERCA2a has been observed to be upregulated by exercise training. This has significant implication with respect to cardiac contractility because SERCA2a regulates the uptake of ${\mathrm{Ca}}^{2+}$ into sarcoplasmic reticulum (SR), and affects cardiac relaxation, ${\mathrm{Ca}}^{2+}$ loading of the SR, and consequently the amount of ${\mathrm{Ca}}^{2+}$ available for release during cardiac myocyte contraction [94, 95]. On top of SERCA2a, cardiac excitation-contraction (E-C) coupling is in fact mainly initiated by ${\mathrm{Ca}}^{2+}$ influx through L-type voltage gated ${\mathrm{Ca}}_{\text{V}}$1.2. Calcium channel (${\mathrm{Ca}}_{\text{V}}$1.2) in cardiomyocytes via ${\mathrm{Ca}}^{2+}$-induced ${\mathrm{Ca}}^{2+}$ release mechanisms [96], and more importantly, the expression of ${\mathrm{Ca}}_{\text{V}}$1.2. ${\mathrm{Ca}}_{\text{V}}$1.2 channels is reduced in TAC induced cardiac pathological hypertrophy and heart failure [97]. Studies showed that exercise could partially affect heart function by altering calcium channel levels and calcium signaling proteins [98, 99].

The heartbeat originates from the sinoatrial node (SA) in the right atrium of the heart. SA acts as the pacemaker and generates regular electrical impulses. Exercise training was found to control the density and activity of several pumps, channels, and processes linked with cardiac action potential (AP) and E-C coupling [100]. In order to meet the energy requirement of exercise, heart rate and myocardial contractility show a corresponding increase [94]. This is a reaction of the autonomic nervous system and hormones, wherein the heart rate increases by acting on SA and enhances the contractility of cardiomyocytes by modulating the components of ion current, pump and E-C coupling [100].

### 4.3 Enhancement of Cardiomyocyte Energy Metabolism

The heart requires a large amount of energy supplement and needs to constantly produce ATP to maintain its contractile function, ion homeostasis, anabolic processes and signaling transduction [101, 102, 103]. The number and size of mitochondria are controlled by the process of mitochondrial fusion and division [104]. Suppressing excessive mitochondrial division is deemed to be good for cardiac function [105]. Exercise training is confirmed to regulate the alterations in fusion and division-related proteins, which can prevent myocardial infarction-induced mitochondrial fusion reduction and division increase [106, 107]. About 60% to 70% ATP is used by the heart to promote contraction, while about 30% to 40% of the remaining ATP is used by various ion pumps, especially ${\mathrm{Ca}}^{2+}$-ATPase in the sarcoplasmic reticulum (SR). Therefore, to a large extent, the cardiac function depends on the production of ATP, and damages in this process will quickly induce contractile dysfunction [108, 109]. During exercise, increased circulation and local production of catecholamines result in raised heart rate and muscle strength, which in turn lead to moderations in cardiac metabolism [110, 111]. Both epinephrine and norepinephrine can promote the oxidation of endogenous triglycerides. Increase in plasma free fatty acid (FFA) levels during exercise adaptation could be considered sufficient to increase myocardial fat catabolism [112].

The upregulation of neuregulin-1 by exercise training can induce interleukin-1α (IL-1α) and interferon-γ (IFN-γ), which are associated with paracrine cardiac cytokines, as well as pro-repair factors such as angiogenin-2, brain-derived neurotrophic factor and crypto-1 [113, 114]. These factors have been shown to contribute to the repair mechanism of the heart. In chronic heart failure, neuregulin-1 has been shown to regulate reverse cardiac remodeling, and it remains elevated during exercise adaptation and further increase glucose absorption and utilization [114]. Other studies have found that exercise training regulates myocardial glycolytic activity due to the expression of kinase 6-phosphofructo-2-kinase/fructose-2,6-bisphosphatase (PFK2) [115, 116]. Insulin-like growth factor 1 (IGF-1) has also been reported to affect cardiac energy requirements and metabolism through its role in muscle strength [47, 59, 108].

**Fig. 2.**
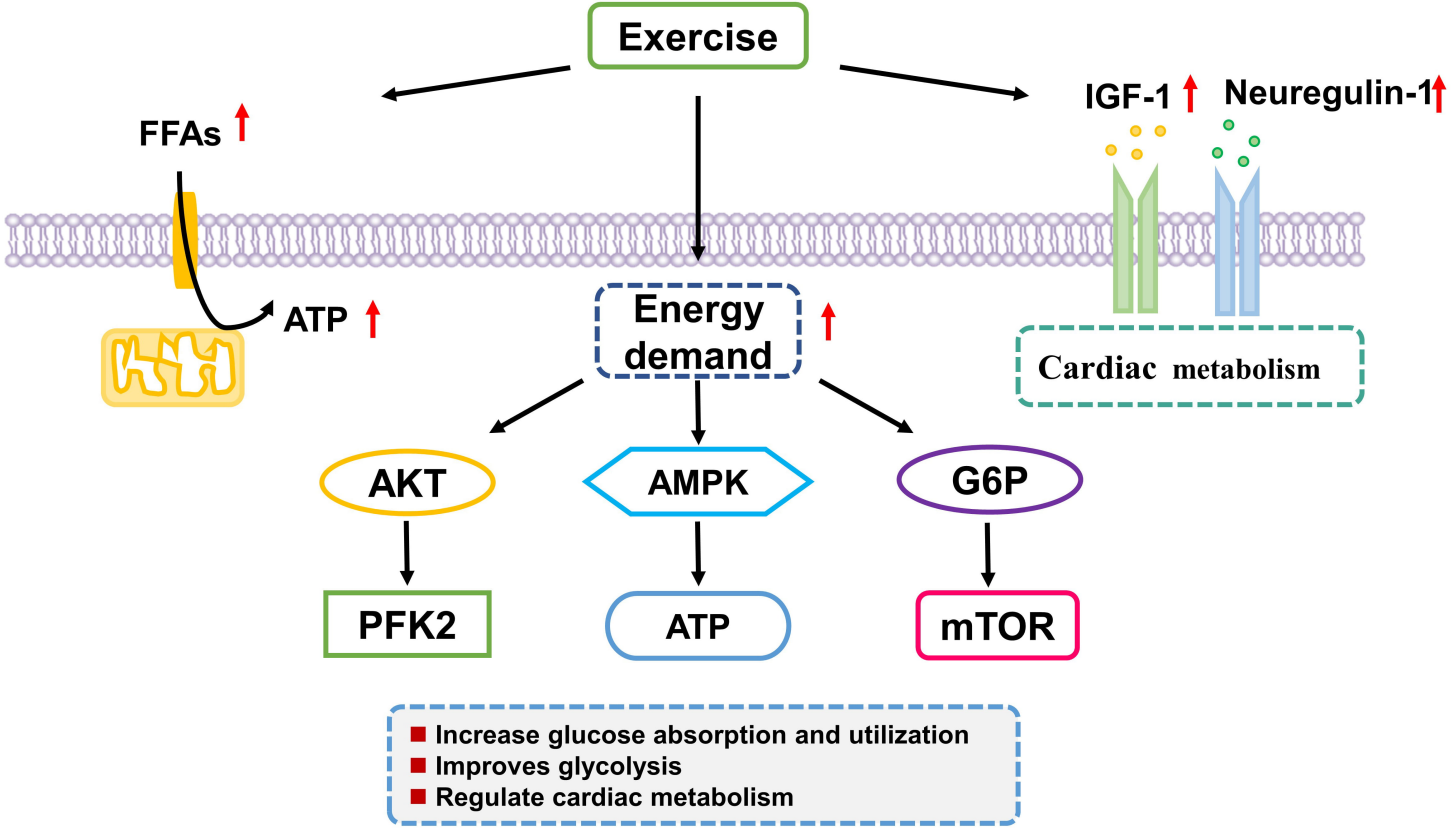
**Exercise training regulates myocardial cells energy metabolism**. Exercise training causes adaptations in energy metabolism of the heart including glucose absorption and utilization and glycolysis. Akt, serine/threonine kinase; FFA, plasma free fatty acid; AMPK, AMP Activated Protein Kinase PFK2, 6-phosphofructo-2-kinase/fructose-2,6-bisphosphatase; ATP, adenosine triphosphate; G6P, glucose-6-phosphate.

Studies have shown that exercise training can increase the level of circulating extracellular vesicles [117, 118]. These vesicles can transfer metabolic enzymes to the recipient cells, thereby changing metabolism of the recipient tissue [119]. The cellular fuel agent AMP-activated protein kinase (AMPK), that senses the levels of AMP and ATP in cells, is activated during exercise. When the energy demand is high, AMPK will be activated to enhance ATP levels by increasing glucose and fatty acid catabolism and simultaneously inhibiting protein synthesis. Metabolites involved in glucose and fat metabolism have also been implicated as regulators of exercise-induced heart growth. In fact, changes in glucose-6-phosphate (G6P) levels in cells has been shown to promote ventricular remodeling by regulating mammalian target of rapamycin (mTOR) signaling [120] (Fig. 2).

### 4.4 Reduction in Oxidative Stress of Cardiomyocytes

**Fig. 3.**
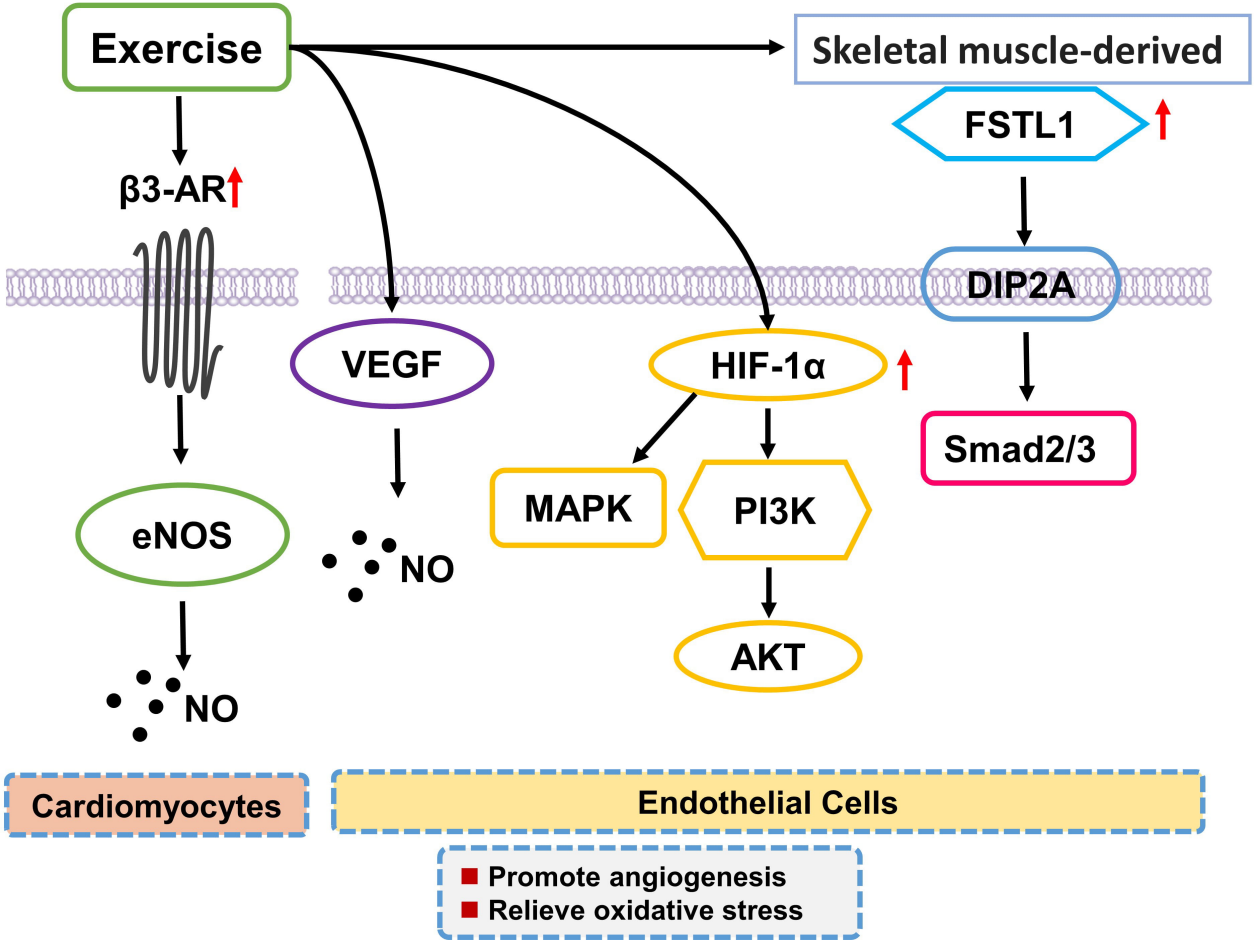
**Exercise training inhibits oxidative stress and promotes angiogenesis**. Exercise training increases the expression of β3-AR after myocardial infarction and relieves oxidative stress of cardiomyocytes by regulating eNOS-NO signaling pathway. Exercise training also regulates the VEGF and NO expression and reverses arterial dysfunction in the endothelial vessel wall. In addition, exercise training upregulates the expression of HIF-1α, resulting in angiogenesis stimulation through PI3K-Akt-eNOS and MAPK signaling pathway. Alternatively, exercise training also stimulates skeletal muscle to secrete FSTL1 and promotes myocardium angiogenesis. β3-AR, β-adrenergic receptors; NOS, endothelial nitric oxide synthase; NO, nitric oxide; HIF-1α, hypoxia-Inducible factor 1-alpha; MAPK, mitogen-activated protein kinase; PI3K, phosphoinositide-3-kinase; VEGF, vascular endothelial growth factor; Akt, serine/threonine kinase; FSTL1, follistain like-1; DIP2A, disco-interacting protein 2 homolog A.

Oxidative stress is the excessive production of reactive oxygen species (ROS) associated with antioxidant defenses, and can affect ventricular remodeling [121]. eNOS, the predominant NOS isoform in vasculature, has a crucial role in many protective effects attributed to exercise. In the presence of its cofactors, electrons from reduced nicotinamide adenine dinucleotide phosphate (NADPH) could be transferred by eNOS to heme site via flavin adenine dinucleotide and flavin mononucleotide. The electrons are used to decrease and activate oxygen and oxidize L-arginine to L-citrulline and NO [122, 123, 124]. After four weeks of random wheel running training in mice, circulating adrenaline and norepinephrine levels were found to be increased, and myocardial eNOS and NO production were consequently activated, bestowing a protective effect on ischemic-reperfusion injury [125, 126]. Similar studies demonstrate that exercise training increases the expression of β3-adrenoceptor agonist (β3-AR) after myocardial infarction and attenuates oxidative stress of cardiomyocytes by regulating eNOS-NO signaling [127] (Fig. 3).

Exercise training also has a beneficial effect in balancing cardiac nitroso redox by activating ROS scavenging enzymes, like superoxide dismutase [128]. In cardiomyocytes, mitochondria can transfer energy between myofibrils by offering ATP to cell membrane ion pumps. Basal ROS levels in cardiomyocytes of exercise trained mice have been shown to be reduced. In order to promote ventricular remodeling, it is essential to maintain mitochondrial membrane potential, reduce the production of mitochondrial ROS, and protect the redox homeostasis [46, 54, 127, 128, 129, 130, 131, 132, 133].

### 4.5 Promotion of Angiogenesis to Protect Against Ventricular Remodeling

After myocardial infarction, poorly adapted left ventricular remodeling could occur due to impaired angiogenesis, which can further promote transition from adaptive myocardial hypertrophy to left ventricular dilation and dysfunction. Exercise training has been demonstrated to activate VEGF dependent angiogenesis pathways and increase VEGF expression in the heart [134, 135, 136, 137]. After myocardial infarction, exercise training can reverse nitric oxide (NO) induced arterial dysfunction in the endothelial vessel wall [134, 138]. NO has several benefits for cardiovascular functions, including vasodilation, inhibition of platelet aggregation and adhesion, reduction of leukocyte and vascular inflammation level, increased angiogenesis, proliferation of vascular smooth muscle cells, and activation of endothelial progenitor cells [139, 140]. Follistain like-1 (FSTL1) has been reported to play an important role in cardiac protection obtained due to exercise training. Resistance exercise stimulates the skeletal muscle to secrete FSTL1, which binds to the disco-interacting protein 2 homolog A (DIP2A) receptor, and through Smad2/3 signaling promotes myocardial angiogenesis in rats with myocardial infarction [141]. In addition, exercise training upregulates the level of hypoxia-Inducible factor 1-alpha (HIF-1α), triggering angiogenesis promotion through the PI3K-Akt-eNOS and MAPK signaling pathway and protects cardiac function after myocardial infarction [142] (Fig. 3).

### 4.6 Inhibition of Inflammation

After myocardial infarction, the myocardium activates the innate immune system to initiate tissue repair mechanisms, corelated with significant increase in the levels of different kinds of pro-inflammatory cytokines [143]. This increase in pro-inflammatory cytokines contributes to cardiac remodeling [144, 145]. Following an acute pro-inflammatory phase there is an anti-inflammatory response that promotes heart repair [146]. However, the spread of the pro-inflammatory response in the myocardium depends upon the diversity and uniqueness of cardiac pressure. If it is not counteracted by the anti-inflammatory mechanism, this prolonged inflammatory response will turn into chronic inflammation [146, 147]. The key feature of this chronic cardiac inflammation is the continued increase in production of pro-inflammatory cytokines in the heart. These pro-inflammatory cytokines have harmful effect on the myocardium and are participated in the transition from myocardial infarction to heart failure [147].

Elevated levels of pro-inflammatory cytokines in circulation and heart are associated with ventricular remodeling, thereby leading to chronic heart failure. Exercise training can inhibit the expression of inflammatory factors, such as TNF-α and IL-6, and increase the abundance of immunosuppressive factor IL-10 [148, 149]. Aerobic exercise training was verified to promote endothelial function through regulation of these mechanisms, such as, reducing the expression of proinflammatory transcription factor nuclear factor kappa B (NF-κB) and mitigation of oxidative stress [150, 151]. Downregulation of Toll-like receptor 4 (TLR4), a transmembrane receptor that can induce the production of inflammatory cytokines, also promotes the anti-inflammatory effects induced by exercise training [152]. In both the aerobic exercise group and TLR4 inhibited mice, the expression levels of pro-inflammatory factors namely IL-1β, IL-6, TLR4, NF-κB, and TNF-α were downregulated, and the mRNA expression levels of the anti-inflammatory factor IL-10 was upregulated [153, 154]. Previous study showed that exercise induced a reduction in TNF-α and IFN-α production in response to R-848 by Toll-like receptor (TLR-7) [61], and TLR-7 deficiency reduced post-MI scar formation and inflammation [155].

Exercise training can not only protect from myocardial infarction by directly regulating the release of inflammatory factors, but also regulate the activity of immune cells that release inflammatory factors [49, 50, 53]. Compared to the non-exercise control group, continuous high-intensity aerobic exercise training increased the production of anti-inflammatory factors and the number of regulatory T cells (Tregs), and weakened the production of the cytokine interferon γ (IFN-γ). In addition, aerobic exercise training also inhibited the proliferation of antigen-specific T lymphocytes and reaction of antigen-specific cluster of differentiation 8+ (CD8+) cytotoxic T lymphocytes [156]. Studies have also verified that even after stopping the exercise training for a few weeks, the effect of previously consistent and regulated exercise training still lingers [49]. In addition, many studies have revealed the molecular mechanisms by which aerobic exercise training can protect the heart and the whole body through many factors, including but not limited to TNF-α, TGF-β, IL-1β, IL-6, osteoprotegerin and leptin [49, 157]. Cells produce changes in the expression levels of these inflammatory factors and thereby exert exercise-driven cardioprotective effects (Table 2, Ref. [46, 60, 61, 138, 143, 144, 145, 146, 147, 148, 149, 150, 151, 152, 153, 154, 155, 156]).

**Table 2.** **Summary of pro- and anti-inflammatory cytokines regulated by exercise training following myocardial infarction**.

Down-regulation	References	Up-regulation	References
Interleukin-1β	[153, 154]	IL-10	[148, 149, 153, 154]
Interleukin-6	[148, 149, 153, 154]	antigen-specific T cells	[156]
Tumor necrosis factor-α	[61, 148, 149, 153, 154]	CD8+ T cells	[156]
Nuclear factor κB	[150, 151, 153, 154]		
Interferon-γ	[156]		
Interferon-α	[60, 61, 155]		
Transforming growth factor-β	[46, 138]		
Toll-like receptors 4	[152]		
Toll-like receptors 7	[155]		
Regulatory T cells	[156]		

However, based on the recent research evidence, the effects of exercise training on the immune system cannot be generalized conclusively. Different studies have showed that the effects of exercise on immune cells are not only inconsistent, sometimes even contradictory, possibly due to differences in test subjects or the intensity of aerobic exercise. Since the metabolism of the body is a complicated process, the effects of exercise training on myocardial infarction are not uniform across various studies [49, 153, 154, 156, 158, 159, 160, 161, 162, 163]. In addition, the different effects brought by exercise training may be caused by different research individuals, different exercise intensity, and different time [164]. Therefore, it is of great relevance to formulate and prescribe appropriate and customized exercise training based on patient history and nature as well as the degree of the heart disease.

## 5. Summary 

**Fig. 4.**
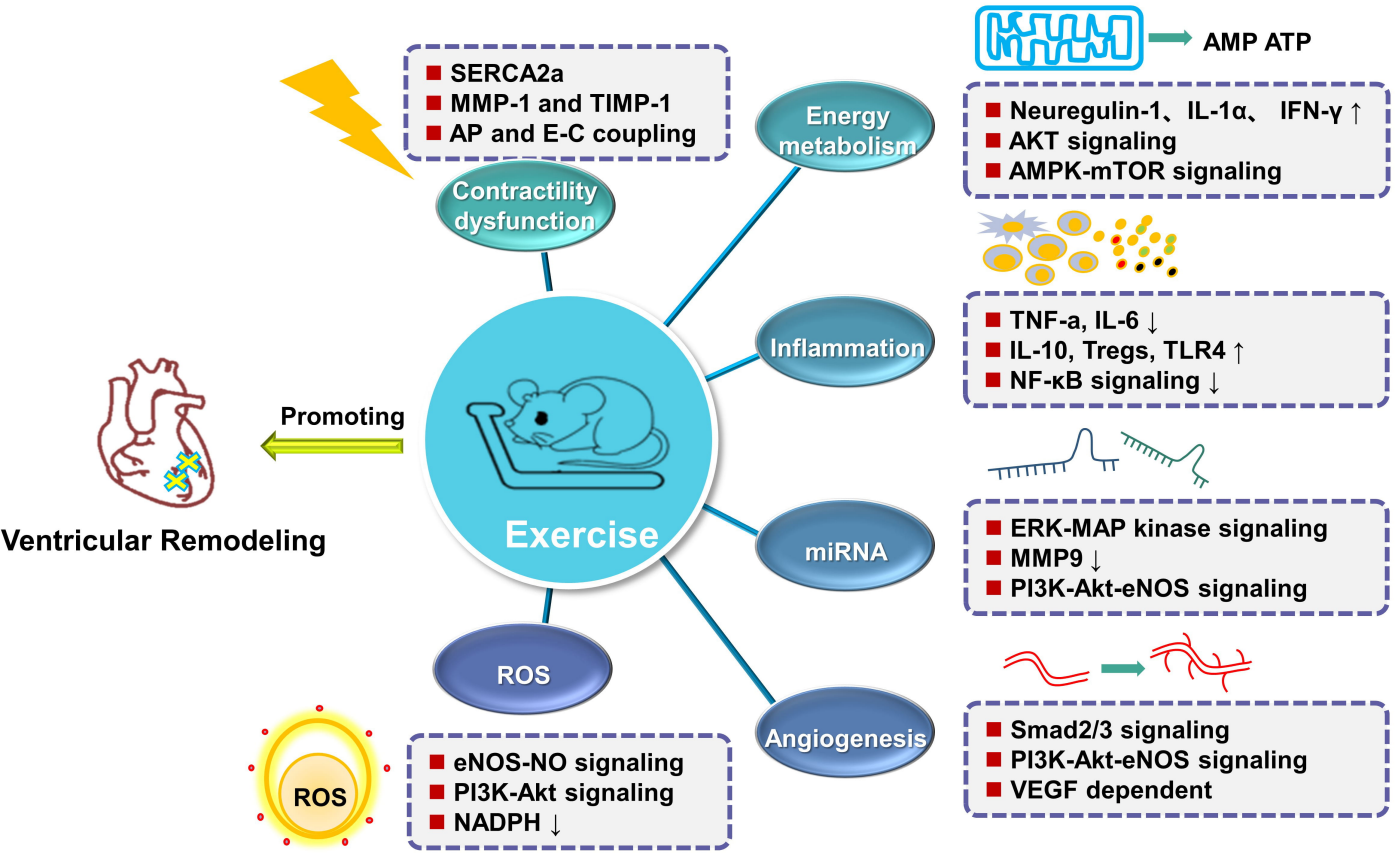
**Summary of protective mechanisms involved in ventricular remodeling observed due to exercise training following myocardial infarction**. Exercise training alleviates ventricular remodeling and restores cardiac function by altering microRNAs expression selectively; adjusting ${\mathrm{Ca}}^{2+}$ homeostasis and collagen accumulation to improve myocardial contractility; regulating energy metabolism of myocardial cells; inhibiting oxidative stress of cardiomyocytes; enhancing VEGF dependent angiogenesis pathways, and/or regulating inflammatory response. VEGF, vascular endothelial growth factor.

Myocardial infarction is considered the most common emergency in cardiovascular system, with high morbidity and mortality, being one of the leading causes for heart failure, causing a great burden on patients and society. Exercise training has a recognized beneficial effect on the heart, irrespective of its healthy or diseased condition. In addition, exercise training becomes one of effective interventions to reverse cardiac remodeling and improve cardiac function in patients with heart failure. Exercise training can reverse ventricular remodeling after myocardial infarction via multiple mechanisms including regulating the expression of miRNA in cardiac tissues [25, 62, 63, 64, 65, 66, 67, 68, 69, 71, 72, 73, 74, 75, 76, 77, 78, 79, 80, 81, 82, 83, 84, 85, 134], enhancing myocardial contractility [20, 82, 86, 87, 88, 89, 90, 91, 92, 93, 94, 95, 96, 97, 98, 99, 100], regulating cardiomyocyte energy metabolism [47, 101, 102, 103, 104, 105, 106, 107, 108, 109, 110, 111, 112, 113, 114, 115, 116, 117, 118, 119, 120], reversing oxidative stress [46, 54, 121, 122, 123, 124, 125, 126, 127, 128, 129, 130, 131, 132, 133], promoting angiogenesis [134, 135, 136, 137, 138, 139, 140, 141, 142] and reducing inflammation [49, 143, 144, 145, 146, 147, 148, 149, 150, 151, 152, 153, 154, 155, 156, 157, 158, 159, 160, 161, 162, 163]. In this review, several recent evidence regarding the mechanisms involved in executing the protective effects of exercise training post-myocardial infarction have been summarized (Fig. 4).

Although regular physical activity reduces cardiovascular disease, vigorous activity also increases the risk of acute myocardial infarction and sudden cardiac death in susceptible individuals [165, 166, 167, 168, 169]. In people with diseased or susceptible hearts, vigorous and high-intensity exercise may increase the risk of worsening cardiovascular function, acute cardiac events, or sudden cardiac death (SCD) in some individuals [170, 171]. There is substantial epidemiological, basic science and clinical evidence that habitual physical activity reduces the risk of cardiovascular disease and that the benefits of regular physical activity outweigh the risks [164, 165, 168]. Research suggests that individuals should do ≥30 minutes of moderate-intensity physical activity each day [172]. Individuals who start exercising should start slowly and increase the intensity and duration of the exercise as their tolerance allows. In addition, exercise should be assessed according to AHA/American College of Cardiology and relevant guidelines [173, 174, 175]. Further studies are required for investigating the role of organ or tissue cross-talk in exercise mediating cardiovascular protection effects. Several studies have shown that exercise has similar functions to many other drugs, such as protecting chronic heart disease and as a treatment for heart failure [176]. Therefore, combined exercise training and heart medication may result in improvements in cardiovascular disease [177]. Although interaction between exercise and anticoagulant, antiplatelet, angiotensin II receptor blockers, calcium channel blockers and statins have been reported to be involved in protecting against cardiovascular disease [176], the critical aspects of exercise-induced cardioprotection may be changed by the complexity of exercise-drug interactions [177, 178]. The combination of drug and exercise can be beneficial in some cases and harmful in others [176, 177]. It is necessary to further study the adverse effects.
